# Screening for Biomarkers Associated with Left Ventricular Function During Follow-up After Acute Coronary Syndrome

**DOI:** 10.1007/s12265-022-10285-2

**Published:** 2022-06-21

**Authors:** Christina Christersson, Tomasz Baron, Frank Flachskampf, Lars Lindhagen, Bertil Lindahl, Agneta Siegbahn

**Affiliations:** 1 Department of Medical Sciences, Cardiology, Uppsala University, 75185 Uppsala, SE Sweden; 2 Uppsala Clinical Research Center, Uppsala University, Uppsala, Sweden; 3 Department of Medical Sciences, Clinical Chemistry, Uppsala University, Uppsala, Sweden

**Keywords:** Soluble biomarkers, Left ventricular function, Acute coronary syndrome, Heart failure

## Abstract

A proportion of patients with the acute coronary syndrome (ACS) will suffer progressive remodeling of the left ventricular (LV). The aim was to screen for important biomarkers from a large-scale protein profiling in 420 ACS patients and define biomarkers associated with reduced LV function early and 1 year after the ACS. Transferrin receptor protein 1 and NT-proBNP were associated with LV function early and after 1 year, whereas osteopontin and soluble ST2 were associated with LV function in the early phase and, tissue-type plasminogen activator after 1 year. Fatty-acid-binding protein and galectin 3 were related to worse GLS but not to LVEF 1 year after the ACS. Proteins involved in remodeling and iron transport in cardiomyocytes were related to worse LV function after ACS. Biomarkers for energy metabolism and fibrosis were exclusively related to worse LV function by GLS. Studies on the functions of these proteins might add knowledge to the biological processes involved in heart failure in long term after ACS.

**Supplementary Information:**

The online version contains supplementary material available at 10.1007/s12265-022-10285-2.

## Introduction

In acute coronary syndrome (ACS), assessment of the left ventricular (LV) function is included in risk evaluation of the patients and forms the base for recommendation of medical treatment and device therapy with intracardiac defibrillator [1]. Echocardiography is the method mostly today used for defining LV function, and ejection fraction (EF) is the most common measure of LV function and determinant of prognosis after ACS [2]. The guideline recommendations of early revascularization with coronary blood flow restoration improve outcomes after ACS and reduce the proportion of patients with impaired LVEF. After the myocardial infarction, with resolution of the initial myocardial stunning, there is an improvement of LVEF in 50% of the patients [3]. However, in a proportion of the patients, there will be a progressive remodeling of the left ventricle contributing to heart failure progression in the long term [4, 5]. After coronary occlusion, the necrotic process in the myocardial cells is triggered by inflammation leading to apoptosis [6]. The inflammatory activation initiated in the myocardium includes infiltration of leukocytes, proliferation of fibroblasts, and release of extracellular matrix proteins [7–10]. There is also evidence of autophagy of the cells which could contribute to a negative remodeling process [11, 12]. The majority of these studies were performed in vitro or in animal models, and our knowledge of how these processes can be evaluated by plasma biomarkers in humans is limited.

In chronic heart failure and also in healthy elderly individuals, several circulating biomarkers have been evaluated as predictors of heart failure hospitalization and mortality [13–16]. N-terminal pro-B-type natriuretic peptide (NT-proBNP) is the biomarker most evaluated and used in clinical practice, even though other markers have been suggested to add information [17]. Increased attention has been paid to soluble biomarkers related to heart function in the field of cardio-oncology to identify individuals with subclinical signs of reduced LVEF due to chemotherapy [18].

There are limitations of LVEF as the only measurement of LV function since it is based on an endocardial measurement and influenced by geometry [19]. Global longitudinal strain (GLS) is an alternative method, reflecting impairment of myocardial deformation [20]. Impaired GLS in ACS patients with preserved LVEF may identify early stages of LV dysfunction and gives additional prognostic information on both reduced and preserved LVEF [21–23]. Biomarkers can be used to broaden the mechanistic insight into LV function after ACS and the aims of this study were to (I) screen for important biomarkers from a large-scale protein profiling in patients with ACS and reduced LV function early and 1 year after the ACS event and (II) describe the biomarkers associated with LVEF and GLS as two methods for evaluation of LV function.

## Methods

### Patient Population

The REBUS (The RElevance of Biomarkers for future risk of thromboembolic events in UnSelected post-myocardial infarction patients) study was a prospective observational study previously published (NCT01102933 ClinicalTrials.gov) [24]. Briefly, during 2010–2012, 421 patients with recent ACS both ST-elevation and non-ST-elevation myocardial infarction (STEMI and NSTEMI) were included 2–5 days after the index event, before discharge from hospital, and followed for 2 years. Information on comorbidities and medical treatment were collected at inclusion and during follow-up. All patients were treated according to international and national guidelines, at the discretion of the responsible physicians. The study was approved by the local ethics committee (Log No. 2009/210) and followed the regulations of the Helsinki Declaration. All patients signed a written informed consent before inclusion.

### Proteomic Profiling

EDTA plasma from inclusion, 2–5 days after the acute ACS, and at 1 year were assessed by Proseek Multiplex CVD III ^96x96^ proximity extension assay (Olink Bioscience, www.olink.com/products/cvd-iii-panel, Uppsala, Sweden), at the Clinical Biomarkers Facility, Science for Life Laboratory, Uppsala University, Uppsala, Sweden [25, 26]. The CVDIII panel measures 92 cardiovascular disease-related biomarkers simultaneously and the panel focuses on high-abundance proteins. The kit is based on the proximity extension assay (PEA) technology, where 92 oligonucleotide-labeled antibody probe pairs are allowed to bind to their respective target present in the sample. The PEA technique has a high specificity and sensitivity [27]. The platform provides normalized protein expression (NPX) data where a high protein value corresponds to a high protein concentration, but not an absolute quantification. Samples from 420 patients were available for analysis.

### Left Ventricular Ejection Fraction and Global Longitudinal Strain

Echocardiography was performed in the cardiac intensive care unit within 72 h and during follow-up at 1 year. The prospectively collected echo data were retrospectively reviewed by experienced echocardiographers. Two-dimensional echocardiography was performed in the standard apical four-, three-, and two-chamber views. EF was assessed using the biplane Simpson’s method. Cut-off values concerning systolic function were based on current echocardiographic recommendations; normal LVEF ≥ 54% in women and ≥ 52% in men [28]. External software, Image Arena V 4.6 Build 4.6.4.10 (TomTec Imaging system, Munich, Germany), was used for all speckle-tracking-based analyses previously described [29]. In all apical views, the endocardial borders were manually traced in the end-systolic frame, while end-diastolic borders were provided automatically with the possibility of manual correction. GLS, expressing longitudinal shortening as a percentage, was then automatically calculated in patients with satisfactory imaging quality at both inclusion and follow-up. Care was taken to ensure a frame rate above 40/s and exams were more than 2 left ventricular segments were not analyzable and were excluded.

### Statistical Methods

Baseline characteristics were presented as median and range for continuous variables, and frequency and percentage for categorical values. A total of 92 biomarkers (CVD III panel) were included in the statistical analyses. The very few missing values (90 for elafin and cathepsin D) were filled by single imputation using chained equations, with age, sex, and all biomarkers as predictors [30]. LVEF and GLS were described as percentage and used as numerical variables. The empirical cumulative distribution function (ECDF) plots describe the biomarkers separated by LVEF and GLS in quartile groups.

Due to a large number of biomarkers, we used various statistical models. For the univariate analyses, considering differences in one biomarker at a time, biomarkers were tested for univariate association with LVEF and GLS using Mann–Whitney tests, correcting for multiplicity using permutation methods [31].

**Fig. 1:**
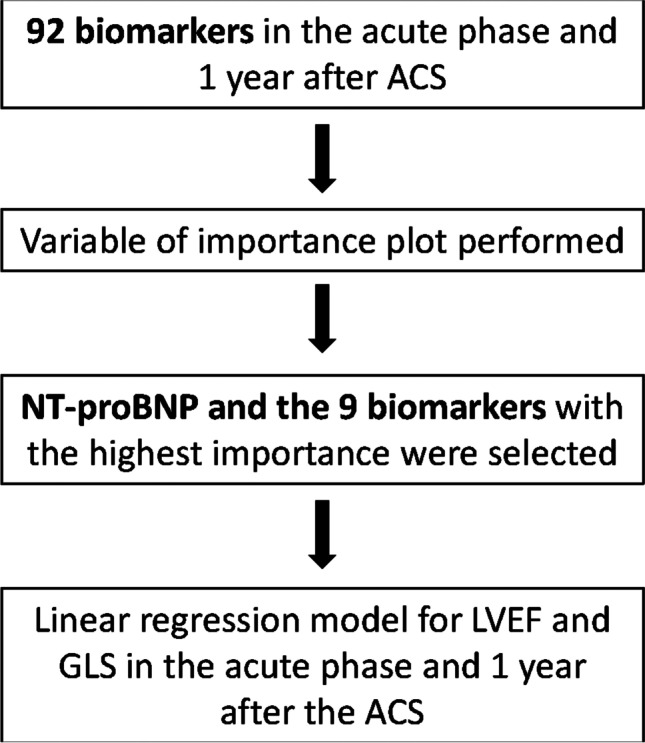
Schematic description of the statistical analyses

In the prediction models of LVEF and GLS, we used all biomarkers simultaneously. Taking into consideration that there are few patients for the number of predictors, standard regression methods are likely to lead to severe overfitting. Instead, we used random forest [32]. This also gives a variable importance plot, ranking the predictors according to how valuable they have been in predicting the outcome, i.e., LVEF or GLS, where 100% variable importance indicates the strongest predictor. These analyses were performed with and without NT-proBNP since this biomarker is a well-known strong predictor of left ventricular function. Based on the results, NT-proBNP and the nine biomarkers identified as variables with the highest importance in the variable plots were selected. These biomarkers were subsequently analyzed one at a time in linear regression models with LVEF or GLS as the dependent variable (Fig. 1). The biomarker was entered as an independent variable in a crude model and in a second model adjusted for age, sex, STEMI, diabetes mellitus, hypertension, and atrial fibrillation. The purpose of the adjusted models was to assess the association between biomarker and LV function that cannot be attributed to clinical background variables. All statistical analyses were performed in R, cf. Section 13.

## Results

### Patient Characteristics

In total 420 patients had available results from the proteomic profiling. The baseline characteristics of these patients are described in Table 1. The median age was 67 (39–95) (min–max) years, 93 (22.1%) were females, and 194 (46.2%) had a STEMI as index ACS. Percutaneous coronary intervention (PCI) was performed, based on clinical decision, in 405 (96.4%), and coronary artery bypass grafting was scheduled in 8 (1.9%) before discharge from the index event. The medical treatment at inclusion is described in Table 2. At discharge, 79.8% of the patients were treated with ACEi/ARB and 92.9% with beta blocking agents. At 1 year after the acute event, 83.6% were prescribed ACEi/ARB and 91.7% beta blocking agents.

**Table 1:** Patient characteristics

	Total cohort, *n* = 420	Females, *n* = 93	Males, *n* = 327
Age*	67 (39–95)	68 (39–92)	66 (39–95)
Smoking current (%)	111 (26.4)	28 (30.1)	83 (25.4)
STEMI^†^ as index MI (%)	194 (46.2)	37 (39.8)	157 (48.0)
Diabetes (%)	68 (16.2)	13 (14.0)	55 (16.8)
Hypertension (%)	224 (53.3)	49 (52.7)	175 (53.5)
Atrial fibrillation (%)	37 (8.8)	8 (8.6)	29 (8.9)

^*^Described as median (min–max)

^†^*STEMI* ST-elevation myocardial infarction

**Table 2:** Medical treatment at inclusion

	Total cohort, *n* = 420	Females, *n* = 93	Males, *n* = 327
Aspirin (%)	412 (98.1)	89 (95.7)	323 (98.8)
ADP-blocking agent (%)	405 (96.4)	87 (93.5)	318 (97.2)
ACEi*/ARB^†^ (%)	335 (79.8)	64 (68.8)	271 (82.9)
Beta blocking agent (%)	390 (92.9)	90 (96.8)	300 (91.7)
Statins (%)	396 (94.3%)	85 (91.4)	311 (95.1)

^*^ACEi angiotensin-converting enzyme inhibitor

^†^*ARB* angiotensin II receptor-blocking agent

### Left Ventricular Function Measurements

Results for LVEF were available in 365 (86.9%) of the patients at inclusion and in 341 (81.2%) 1 year after the ACS. GLS was calculated in 287 out of 365 with results of LVEF (78.6%) at inclusion and 1 year (84.2%). At inclusion, LVEF was in median (min–max) 55 (16–81) % and GLS was − 15.1 (− 24.7– − 4.7) % (Table 3). One year after, the ACS LVEF was in median (min–max) 60 (13–77) % and GLS was − 17.2 (− 25.4– − 5.2) %.

**Table 3:** Left ventricular function in the acute and stable phase after acute coronary syndrome

	Total cohort Median (range) Mean (SD)	Females Median (range) Mean (SD)	Males Median (range) Mean (SD)
LVEF^‡^ at inclusion (%)* (*n* = 365)	59 (16–81) 58 (11)	61 (35–81) 59 (10)	59 (16–80) 57 (11)
LVEF^‡^ at 1 year (%)* (*n* = 341)	60 (13–77) 58 (10)	60 (35–77) 59 (9)	60 (13–75) 57 (10)
GLS^β^ at inclusion* (%) (*n* = 287)	− 15.1 (− 24.7– − 4.7) − 14.8 (4.1)	− 16.4 (− 23.2– − 4.7) − 15.5 (4.3)	− 14.8 (− 24.7– − 5.4) − 14.6 (4.1)
GLS^β^ at 1 year* (%) (*n* = 287)	− 17.2 (− 25.4– − 5.2) − 16.7 (4.1)	− 16.7 (− 24.2– − 6.0) − 16.5 (3.7)	− 17.5 (− 25.4– − 5.2) − 16.7 (4.2)

^‡^*LVEF* left ventricular ejection fraction

^β^*GLS* global longitudinal strain

### Protein Biomarkers, Left Ventricular Ejection Fraction, and Global Longitudinal Strain in the Acute Phase After the Acute Coronary Syndrome

**Fig. 2:**
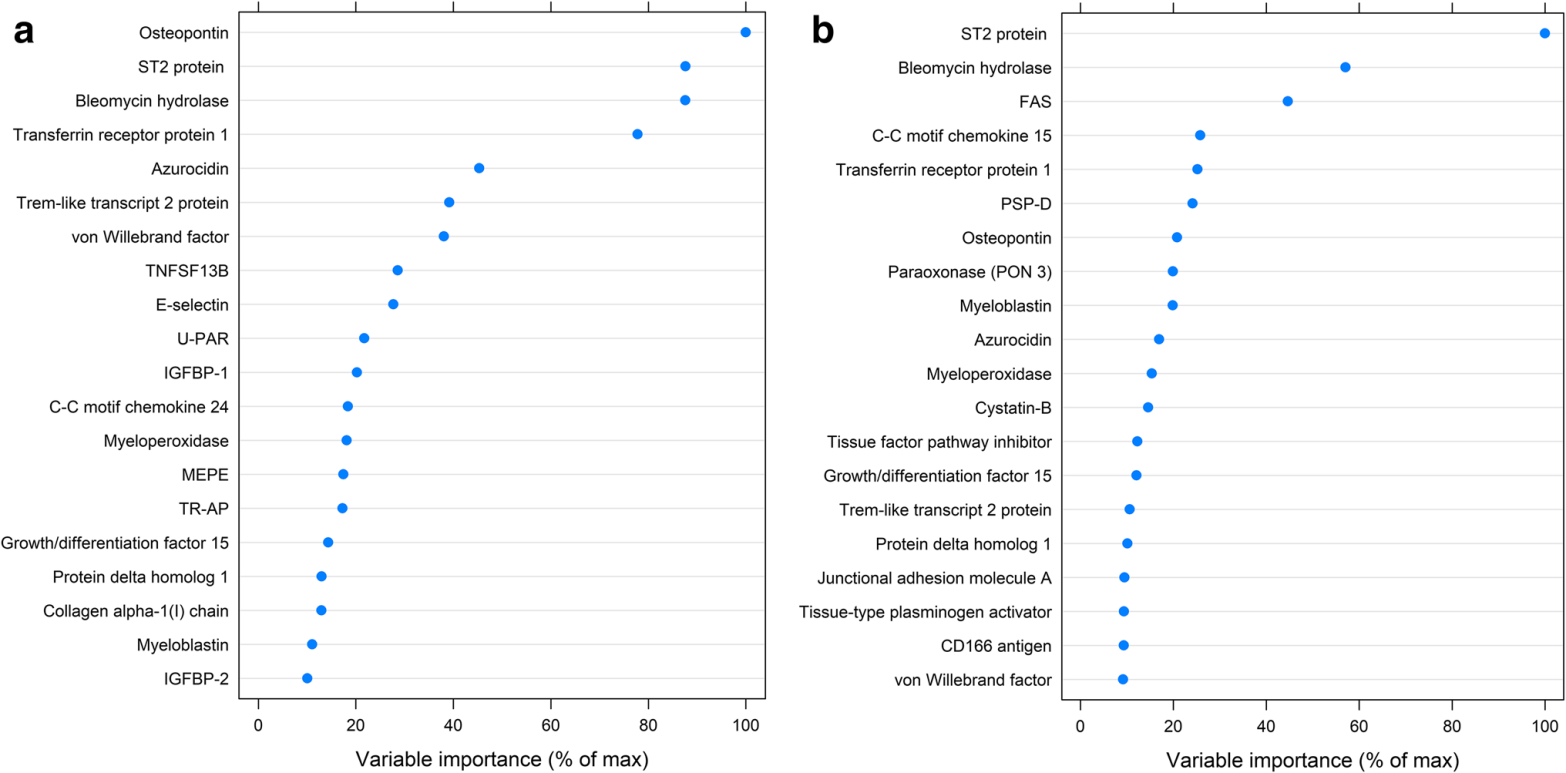
The random forest variable importance plot describes the 20 most important biomarkers for LV function in the acute phase after acute coronary syndrome. The analyses were performed after the exclusion of NT-proBNP. LV function measured as left ventricular ejection fraction (LVEF) (**a**) and global longitudinal strain (GLS) (**b**)

There was a global difference in the analyzed biomarkers associated with LVEF and GLS at inclusion (*p* < 0.0001 for both). In the permutation tests, several biomarkers were identified as associated with LVEF, but only a minor proportion with GLS (Supplement, Fig. 1a–b). After using different methods for analyzing the associations of biomarkers to LVEF, taking into account the multiplicity of using 92 biomarkers at the time, NT-proBNP was the biomarker most pronouncedly associated with LVEF. After excluding NT-proBNP, osteopontin, soluble ST2, bleomycin hydrolase, and transferrin receptor protein 1 were identified as the most important biomarkers associated with LVEF in the acute phase after the ACS (Fig. 2a). Based on these results, the 10 biomarkers with the most importance in the variable plot were further analyzed as independent variables for the outcome LVEF. In the linear regression model, after adjustment for clinical variables, osteopontin had a slope of − 5.25 (95% C.I. − 6.90, − 3.60) for lower LVEF. Transferrin receptor protein 1, tumor necrosis factor ligand superfamily member 13B (TNFSF13B), azurocidin, and von Willebrand factor were also associated with lower LVEF. Similar results were found for the more established markers for heart failure, i.e., NT-proBNP and soluble ST2 (Table 4). The ECDF plots describing the cumulative distribution function for these biomarkers for LVEF quartiles are described in supplement Fig. 2.

**Table 4:** Biomarkers associated with left ventricular ejection fraction (LVEF) in the acute phase and 1 year after acute coronary syndrome (ACS)

Biomarker	Random forest position	Unadjusted slope (95% C.I.)	Adjusted* slope (95% C.I.)
LVEF, acute phase after ACS			
N-terminal prohormone brain natriuretic peptide (NT-proBNP)	*x*	− 4.39 (− 5.20, − 3.59)	− 4.85 (− 5.74, − 3.95)
Osteopontin	1	− 5.27 (− 6.86, − 3.68)	− 5.25 (− 6.90, − 3.60)
ST2 protein	2	− 4.57 (− 5.91, − 3.23)	− 3.84 (− 5.22, − 2.46)
Bleomycin hydrolase	3	− 6.36 (− 8.98, − 3.74)	− 5.92 (− 8.48, − 3.35)
Transferrin receptor protein 1	4	− 4.21 (− 5.96, − 2.46)	− 4.01 (− 5.73, − 2.30)
Azurocidin	5	− 2.42 (− 3.97, − 0.87)	− 2.39 (− 3.90, − 0.88)
Trem-like transcript 2 protein	6	1.53 (− 0.77, 3.83)	1.22 (− 1.02, 3.46)
Von Willebrand factor	7	− 2.70 (− 4.02, − 1.39)	− 2.53 (− 3.87, − 1.19)
Tumor necrosis factor ligand superfamily member 13B (TNFSF13B)	8	− 5.09 (− 7.43, − 2.74)	− 5.07 (− 7.37, − 2.77)
E-selectin	9	− 1.65 (− 3.58, 0.29)	− 1.28 (− 3.22, 0.66)
LVEF, 1 year after ACS			
N-terminal prohormone brain natriuretic peptide (NT-proBNP)	*x*	− 3.59 (− 4.61, − 2.58)	− 3.87 (− 5.04, − 2.71)
Tissue-type plasminogen activator	1	− 2.10 (− 3.74, − 0.46)	− 1.81 (− 3.43, − 0.19)
Chitinase 3-like protein	2	0.15 (− 0.87, 1.17)	0.57 (− 0.51, 1.65)
Urokinase plasminogen activator surface receptor (U-PAR)	3	− 2.20 (− 4.24, − 0.16)	− 1.65 (− 3.91, 0.61)
Growth differentiation factor 15 (GDF15)	4	− 1.63 (− 3.12, − 0.15)	− 0.58 (− 2.50, 1.34)
Lymphotoxin beta receptor	5	− 0.11 (− 2.51, 2.28)	0.59 (− 1.97, 3.15)
Paraoxonase 3 (PON 3)	6	2.24 (0.60, 3.88)	1.43 (− 0.24, 3.11)
Tyrosine protein kinase receptor UFO (AXL)	7	1.69 (− 1.07, 4.45)	1.92 (− 0.78, 4.63)
Insulin-like growth factor-binding protein 7 (IGFBP-7)	8	− 3.10 (− 5.45, − 0.76)	− 2.42 (− 4.89, 0.06)
Transferrin receptor protein 1	9	− 0.69 (− 2.36, 0.99)	− 0.46 (− 2.14, 1.23)

^*^Adjustment for age, sex, STEMI, diabetes mellitus, hypertension, and atrial fibrillation

The same analyses were performed for the outcome GLS (Fig. 2b). In the linear regression model, after adjustment for clinical variables, bleomycin hydrolase had a slope of 2.80 (95% C.I. 1.73–3.87) for reduced GLS. Transferrin receptor protein 1, osteopontin, myeloblastin, cc-motif chemokine 15, and pulmonary surfactant-associated protein D, were also associated with reduced GLS (Table 5). Of the more established biomarkers, NT-proBNP and soluble ST2 were associated with reduced GLS in the acute phase after the ACS. The ECDF plots for these biomarkers in the GLS quartiles are described in supplement Fig. 3.

**Table 5:** Biomarkers associated with global longitudinal strain (GLS) in the acute phase and 1 year after acute coronary syndrome (ACS)

Biomarker	Random forest position	Unadjusted slope (95% C.I.)	Adjusted* slope (95% C.I.)
GLS, acute phase after ACS			
N-terminal prohormone brain natriuretic peptide (ntproBNP)	*x*	1.77 (1.40, 2.14)	1.64 (1.23, 2.06)
ST2 protein	1	1.71 (1.12, 2.30)	1.23 (0.65, 1.81)
Bleomycin hydrolase	2	2.98 (1.84, 4.12)	2.80 (1.73, 3.87)
Tumor necrosis factor receptor super family member 6 (FAS)	3	0.09 (− 1.02, 0.83)	0.14 (− 0.75, 1.02)
c–c motif chemokine 15	4	1.23 (0.48, 1.98)	0.94 (0.20, 1.68)
Transferrin receptor protein 1	5	1.49 (0.71, 2.27)	1.41 (0.68, 2.15)
Pulmonary surfactant-assoc. Protein D (PSP-D)	6	0.97 (0.39–1.56)	0.76 (0.20–1.32)
Osteopontin	7	1.65 (0.92, 2.39)	1.13 (0.38, 1.87)
Paraoxonase 3 (PON 3)	8	0.66 (− 1.35, 0.02)	− 0.44 (− 1.11, 0.23)
Myeloblastin	9	1.73 (0.95, 2.52)	1.49 (0.75, 2.23)
GLS, 1 year after ACS			
N-terminal prohormone brain natriuretic peptide (ntproBNP)	x	1.46 (0.96, 1.96)	1.17 (0.60, 1.74)
Cystatin-B	1	1.27 (0.51, 2.04)	0.77 (− 0.04, 1.58)
Fatty-acid-binding protein, adipocyte (FABP 4)	2	0.92 (0.36, 1.47)	0.71 (0.12, 1.29)
Paraoxonase 3 (PON 3)	3	− 0.99 (− 1.70, − 0.27)	− 0.64 (− 1.34, 0.07)
Transferrin receptor protein 1	4	0.90 (0.17, 1.63)	0.83 (0.12, 1.53)
Collagen alpha 1 chain	5	− 0.55 (− 1.68, 0.58)	− 0.68 (− 1.76, 0.40)
Tumor necrosis factor ligand superfamily member 13B (TNFSF13B)	6	0.57 (− 0.50, 1.64)	0.16 (− 0.89, 1.21)
Galectin 3	7	1.46 (0.38, 2.53)	1.19 (0.13, 2.24)
Tissue-type plasminogen activator	8	1.04 (0.32, 1.76)	0.80 (0.12, 1.49)
Epithelial cell adhesion molecule	9	− 0.19 (− 0.64, 0.26)	− 0.14 (− 0.58, 29)

^*^Adjustment for age, sex, STEMI, diabetes mellitus, hypertension, and atrial fibrillation

### Protein Biomarkers, Left Ventricular Ejection Fraction, and Global Longitudinal Strain 1 Year After the Acute Coronary Syndrome

**Fig. 3:**
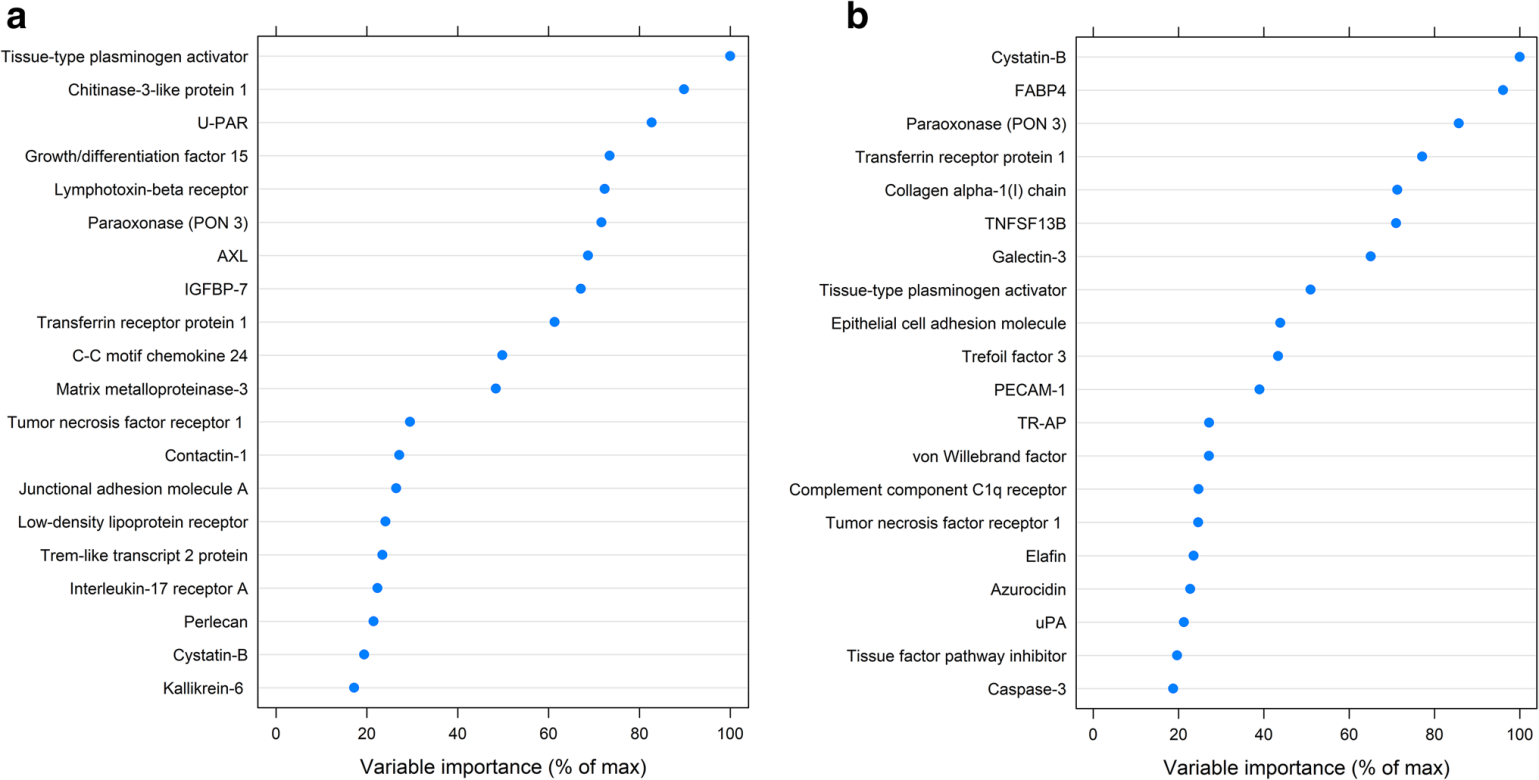
The random forest variable importance plot describes the 20 most important biomarkers for LV function 1 year after acute coronary syndrome. The analyses were performed after the exclusion of NT-proBNP. LV function is measured as left ventricular ejection fraction (LVEF) (**a**) and global longitudinal strain (GLS) (**b**)

There was a global difference in the biomarkers analyzed associated with LVEF and GLS at 1 year (*p* < 0.0001 for both) (Supplement, Fig. 4a–b). The 10 biomarkers with the most importance in the variable plot were further analyzed as independent variables for the outcome LVEF (Fig. 3a). In the linear regression model, after adjustment for clinical variables, tissue factor plasminogen activator had a slope of − 1.81 (95% C.I. − 3.43, − 0.19) and NT-proBNP had a slope of − 3.87 (95% C.I. − 5.04, − 2.71) for lower LVEF (Table 3). The association of urokinase plasminogen activator surface receptor (U-PAR), insulin-like growth factor binding protein 7 (IGFBP7), growth differentiation factor 15 (GDF15), and paraoxonase 3 (PON3) and LVEF attenuated after adjustment for clinical variables (Fig. 3a and Table 4). ECDF plots for the biomarkers separated in LVEF quartiles are described in supplement Fig. 5.

At 1 year after the index ACS, galectin 3 (slope 1.19 (95% C.I. 0.13, 1.21)) and NT-proBNP (slope 1.17 (95% C.I. 0.60, 1.74)) were the biomarkers with the highest association to worse GLS(Fig. 3b and Table 5). Transferrin receptor protein 1, tissue-type plasminogen activator, and fatty-acid-binding protein were also found related to worse GLS, after adjustment for clinical variables. The association of cystatin-B and paraoxonase 3 and GLS attenuated after adjustment for clinical variables (Fig. 3b and Table 5) The ECDF plots for the biomarkers separated in GLS quartiles are described in supplement Fig. 6.

## Discussion

In the present study, we explored the associations of a large group of soluble biomarkers to the LV function in the acute phase and 1 year after ACS. We found a different protein profile associated with the acute phase compared to a more stable phase of reduced LV function where bleomycin hydroxylase, soluble ST2, and osteopontin were found related to LV function only in the acute phase, and tissue-type plasminogen activator in the stable phase. In contrast, transferrin receptor protein 1, as well as NT-proBNP, was associated with LV function both in the acute and stable phases. There was also a different association of biomarkers to LV function when the LVEF echo technique compared to GLS was used as the method to define function.

Biomarkers established as markers for heart failure and used in clinical practice, such as NT-proBNP and soluble ST2, were associated both when LVEF and GLS were used to define LV function. GLS has previously been described as associated with NT-proBNP in patients with preserved ejection fraction, which was confirmed in the present study including both patients with reduced and preserved ejection fraction [33]. The association of soluble ST2 and LV function was only observed in the acute phase, and we could not confirm the findings 1 year after the acute ACS [34].

### Soluble Biomarkers Reflecting Cardiomyocyte Performance and LV Function

We found several new proteins associated with LV function in patients with ACS. Osteopontin is a protein with diverse functions and is suggested to be involved in the interplay of extracellular matrix and cardiomyocytes, important for remodeling affecting both apoptosis and necrosis [35]. It has previously been found increased in animal models with myocardial hypertrophy and in myocardial biopsies in patients with dilated cardiomyopathy [36, 37]. In the present study, osteopontin was associated with worse LVEF and GLS in the acute phase, which might indicate an ongoing myocardial remodeling process in patients early after ACS. In a small study of patients with myocardial infarction and also in patients with chronic heart failure, osteopontin identifies the group of patients with higher risk of mortality [38–40].

Transferrin receptor protein 1 is the main protein for iron transport into the cardiomyocytes. The iron hemostasis within cardiomyocytes is complex and not yet fully understood and both depletion and overload of iron can affect LV function [41]. The transferrin receptor protein 1 was associated with the LV function, both measured as LVEF and GLS, in the acute phase which is in accordance with previous studies. Upon reperfusion injury, the mitochondria induce production of free radicals in a way that involves iron, and high concentrations of the transferrin receptor protein 1 are associated with worse outcome in acute heart failure patients [42–44]. Iron deposition due to intra-myocardial hemorrhages after ACS are also a factor contributing to worse outcome [45, 46]. The association of the transferrin receptor protein 1 and LV function was also found when measured in a stable phase, 1 year after the ACS. The mechanisms behind this finding are today not understood, but it has been described that iron deposition in myocardium from patients with heart transplantation is negatively correlated to the soluble transferrin receptor protein 1 [47].

Paraoxonase 3 was associated with LV function in the stable phase, but the association attenuated after adjustment for clinical variables. In animal studies, paraoxonase 3 is upregulated in the cause of cardiomyocyte remodeling [48]. The knowledge of paraoxonase 3 in human is limited but it has been found associated with iron deficiency in patients with worsening heart failure [49].

### Hemostatic and Inflammatory Soluble Biomarkers and LV Function

The hemostatic soluble biomarkers associated with LV function in the present study represented markers of fibrinolysis. Soluble U-PAR was related to LVEF in the stable phase after ACS. It regulates monocyte adhesion in myocardial infarction and correlates with future risk of heart failure [50, 51]. Tissue-type plasminogen activator was also associated with LV function in the stable phase, measured both with LVEF and GLS. Our knowledge of these fibrinolytic biomarkers in the context of heart failure is limited, but some studies have defined them as predictors of adverse events in chronic heart failure patients, suggesting that they reflect the thrombotic state in heart failure [52–54]. In more severe coronary artery disease and in acute decompensated heart failure with endothelial activation, there is an increase in von Willebrand factor, which could contribute to our findings that higher von Willebrand factor was found with lower LVEF in the acute phase of the ACS [55].

Several biomarkers reflecting an immune and inflammatory response, i.e., bleomycin, myeloblastin, azurocidin, TNFSF13B, c–c motif chemokine 15, and pulmonary surfactant-associated protein D, were found associated with LVEF and/or GLS in the acute phase after ACS. These biomarkers can be released in the infarction area, regulate fibrosis, induce apoptosis, and promote wound healing, all processes important in the acute situation but of minor importance in the stable phase 1 year after ACS [56, 57].

### Soluble biomarkers and GLS

When LV function was analyzed 1 year after the ACS, some biomarkers were found associated with GLS but not LVEF. Fatty-acid-binding protein acts as a transport protein and is involved in energy hemostasis released upon myocardial injury, but the increased concentration is transient. In experimental models, higher concentrations of fatty-acid-binding protein can inhibit cell proliferation of cardiomyocytes and induce apoptosis regulated by miR-1 [58, 59]. Galectin 3 is actively involved in myocardial fibrosis-inducing fibroblast proliferation and collagen deposition, mainly studied in animal models [60]. GLS is suggested as a sensitive marker indicating deformation changes of the LV, and when it is used as a measurement of LV function, it adds information, especially in the group of patients with normal or mildly reduced LVEF. Further studies of biomarkers associated with impairment of GLS will add information on whether they reflect processes involved in myocardial deformation.

### Limitations

There are several limitations of the present exploratory study. The sample size of the study cohort is limited, and all patients were recruited at the same university hospital. However, the study cohort was representative of patients included in the national registry during the same time period. The CVD III panel does not include troponin, and since troponin is associated with the size of myocardial damage in ACS, this might have changed the present results. Larger prospective studies with biomarkers as a primary endpoint are needed to confirm our findings. GLS was not measured in the total study cohort, which might have influenced the results. The PEA technique does not permit absolute quantification of the proteins, and therefore clinically relevant cut-off values cannot be defined.

## Conclusion

In conclusion, several proteins reflecting different pathways involved in the complex response to ACS were found associated with LV function measured as LVEF and as GLS.

Osteopontin and transferrin receptor protein 1 are novel biomarkers, with described pathophysiological myocardial interactions, with a clear association to both LVEF and GLS in the acute phase and as well as in the stable phase 1 year after the ACS. Proteins such as galectin 3 and fatty-acid-binding protein were only associated with GLS, and as proteins involved in apoptosis and fibrosis, they might indicate myocardial deformation. The present study emphasizes the importance of further phenotyping patients with heart failure when exploring new biomarkers both regarding the underlying disease mechanisms and how to define LV function.

## Clinical Relevance

By exploring a large number of soluble biomarkers, not used in clinical practice today, new biological processes associated with LV function can be defined. This will contribute to broadening the knowledge of mechanisms involved in heart failure.

## Supplementary Information

Below is the link to the electronic supplementary material.

Supplementary file1 (DOCX 1120 KB)

## Abbreviations

ACEi: Angiotensin-converting enzyme inhibitor/
ACS: Acute coronary syndrome
ARB: Angiotensin II receptor-blocking agent
ECDF: Empirical cumulative distribution function
GLS: Global longitudinal strain
LV: Left ventricular
LVEF: Left ventricular ejection fraction
PCI: Percutaneous coronary intervention
STEMI: ST-elevation myocardial infarction
