# Supplementary material for: Screening for Biomarkers Associated with Left Ventricular Function During Follow-up After Acute Coronary Syndrome
Source: J Cardiovasc Transl Res. 2022 Jun 21;16(1):244–54. doi: 10.1007/s12265-022-10285-2 (PMC9944718; doi:10.1007/s12265-022-10285-2)
Supplement: Supplementary file 1 — Supplementary file1 (DOCX 1120 KB) [file 12265_2022_10285_MOESM1_ESM.docx]

**Supplement material**

**Figure 1 a**


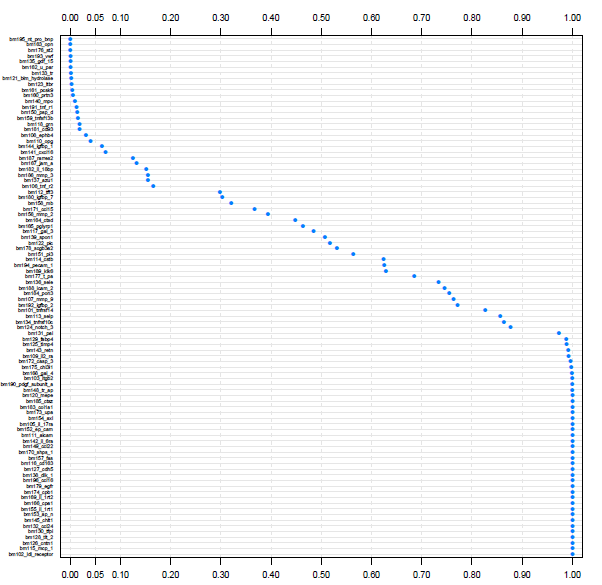


P-value

Biomarkers associated with left ventricular ejection fraction (LVEF) in the acute phase after acute coronary syndrome. Univariate analyses based on permutation tests.

**Figure 1b**


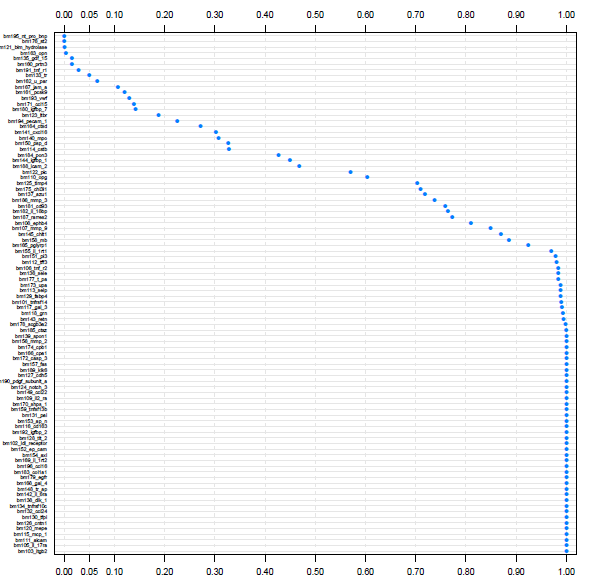


P-value

Biomarkers associated with global longitudinal strain (GLS) in the acute phase after acute coronary syndrome. Univariate analyses of based on permutation tests.

**Figure 2**

**
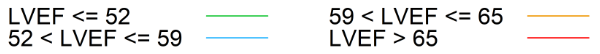
**

**
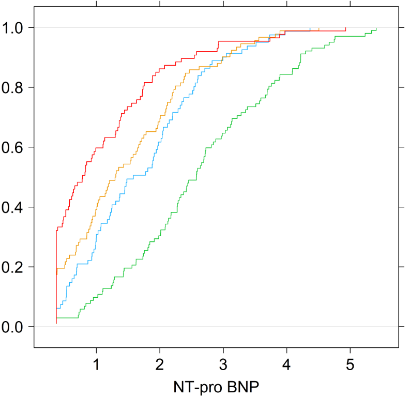

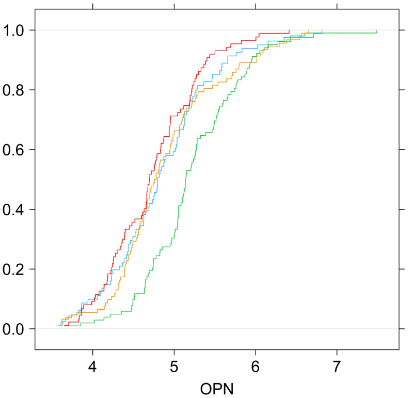

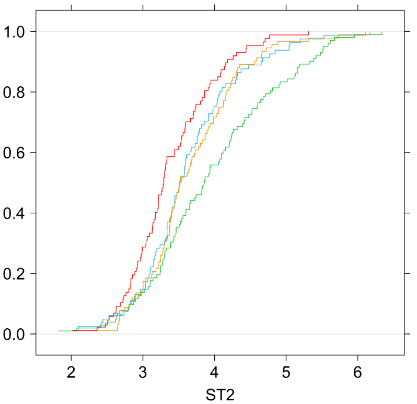
**

**
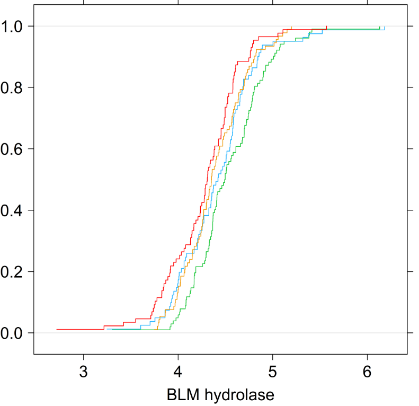

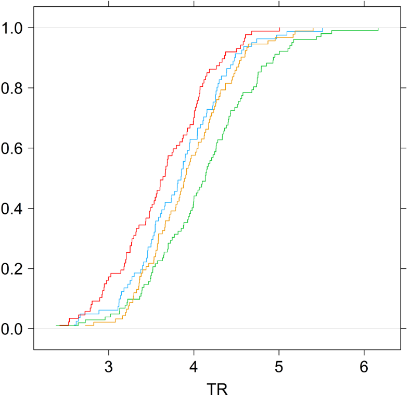

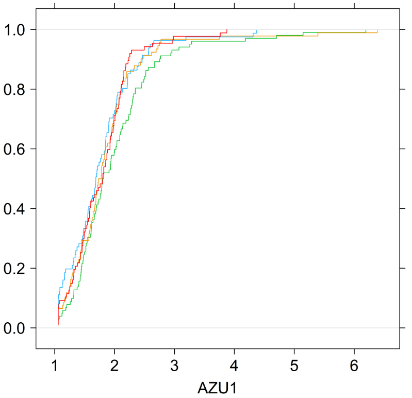
**

**
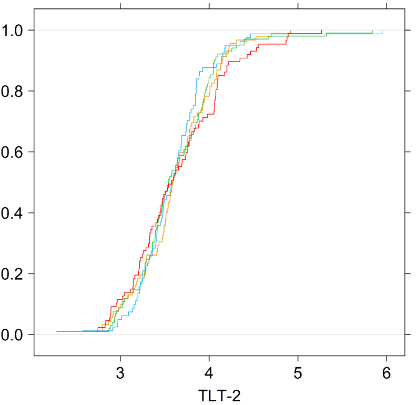

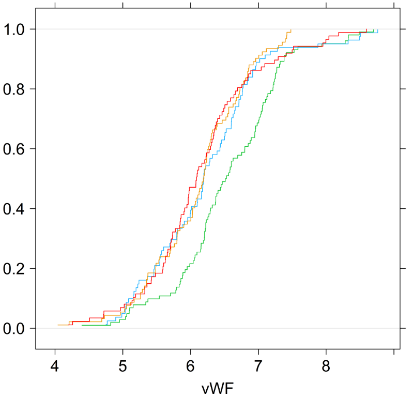

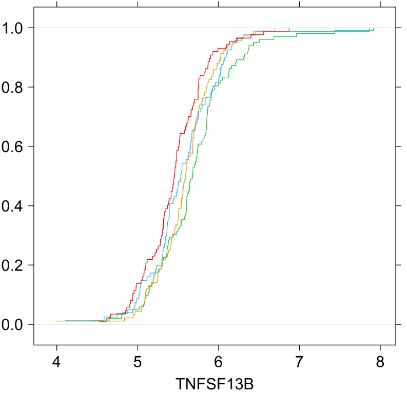
**

**
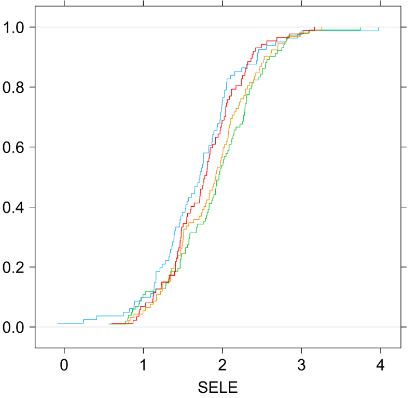
**

OPN; osteopontin, BLM; bleomycin, TR; transferrin receptor protein 1, AZU1; azurocidin, TLT-2; trem-like transcript 2 protein, vWF; von Willebrand factor, TNFSF13B; tumor necrosis factor ligand superfamily member 13B, SEL; E-selectin

The empirical cumulative distribution function (ECDF) plot describing the distribution of the biomarkers expressed as normalized protein expression (NPX) separated by left ventricular ejection fraction (LVEF) quartiles, ≤52% (green), >52%-≤59% (blue), >59%-≤65% (yellow), and >65% (red), in the acute phase after the acute coronary syndrome.

**Figure 3**

**
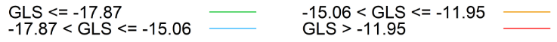
**

**
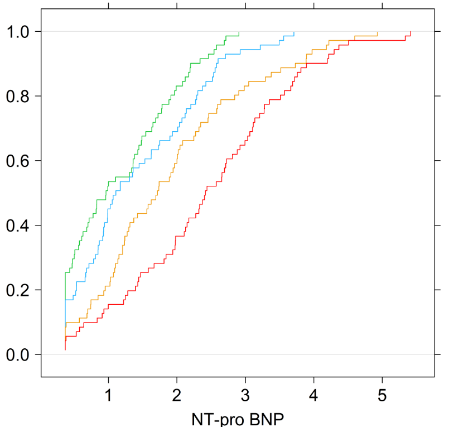

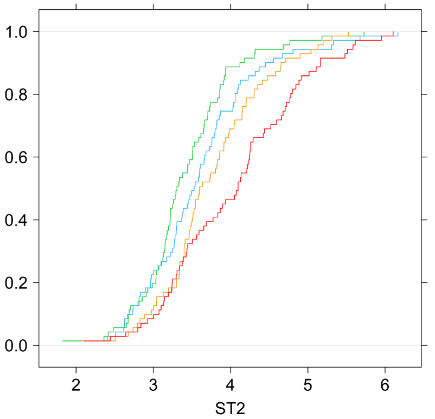

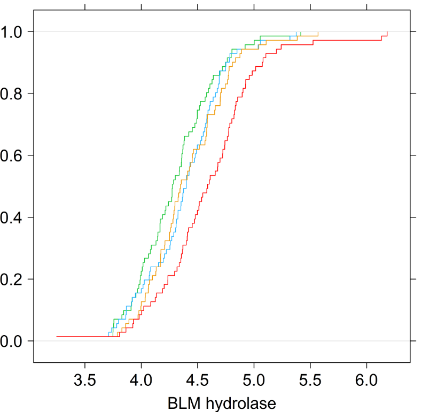
**

**
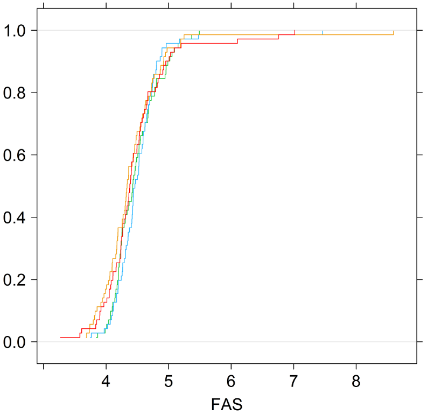

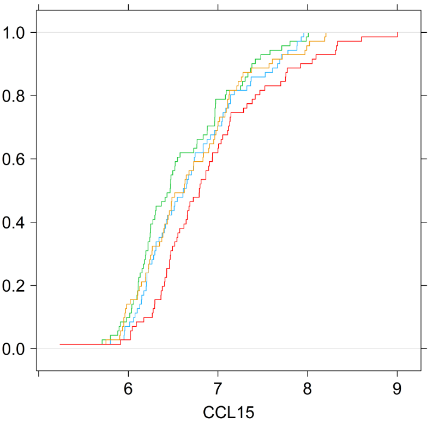

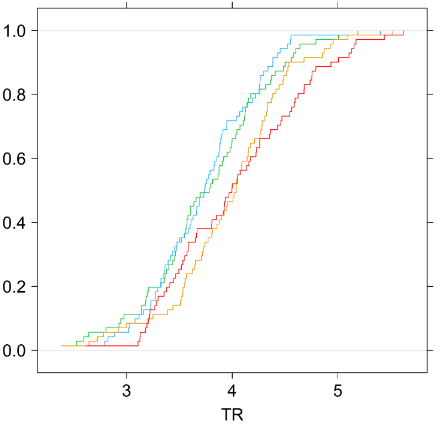
**

**
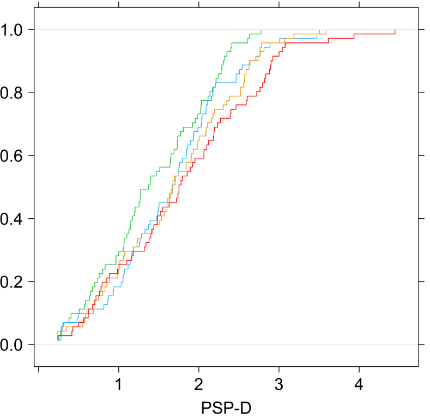

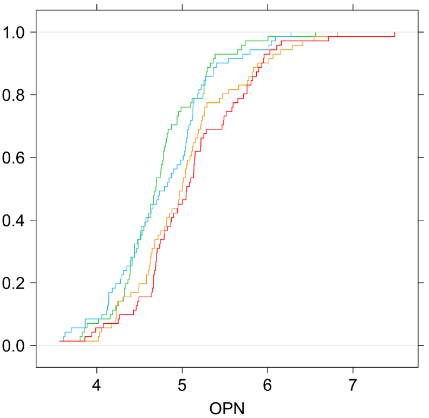

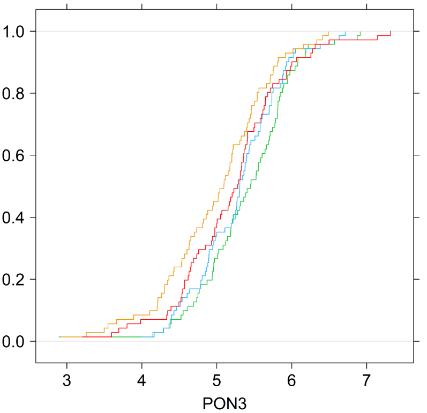
**

**
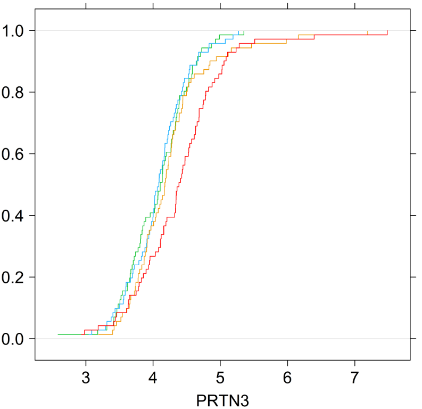
**

BLM; bleomycin, FAS; tumor necrosis factor receptor super family member 6, CCL15; c-c motif chemokine 15, TR; transferrin receptor protein 1, PSP-D; pulmonary surfactant-assoc.protein-D, OPN; osteopontin, PON3; paraoxonase 3, PRTN3; myeloblastin

The empirical cumulative distribution function (ECDF) plot describing the distribution of the biomarkers expressed as normalized protein expression (NPX) separated by global longitudinal strain (GLS) quartiles, ≤- 17.87 % (green), -17.87- ≤-15.06 % (blue), -15.06-≤-11.95 % (yellow) and > -11.95% (red), in the acute phase after the acute coronary syndrome.

**Figure 4a**


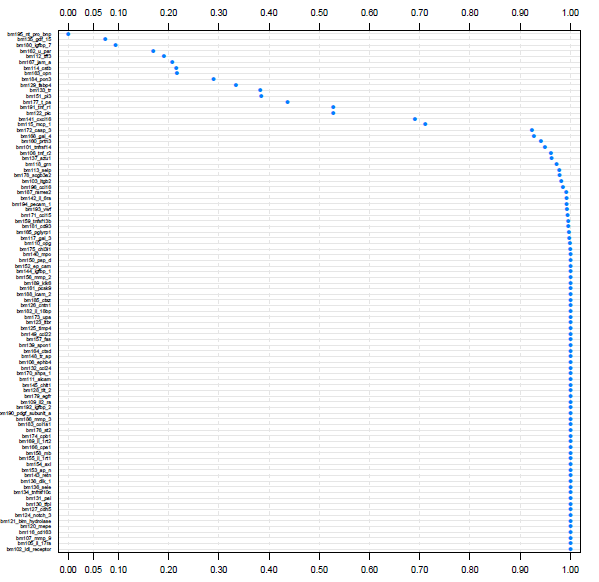


P-value

Biomarkers associated with left ventricular ejection fraction (LVEF) one year after the acute coronary syndrome. Univariate analyses based on permutation tests.

**Figure 4B**


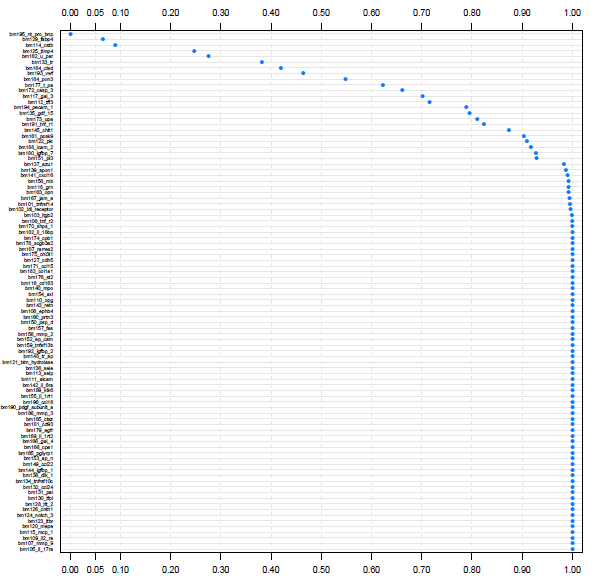


P-value

Biomarkers associated with global longitudinal strain (GLS) one year after the acute coronary syndrome. Univariate analyses of based on permutation tests.

**Figure 5**

**
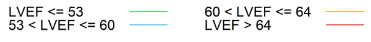
**

**
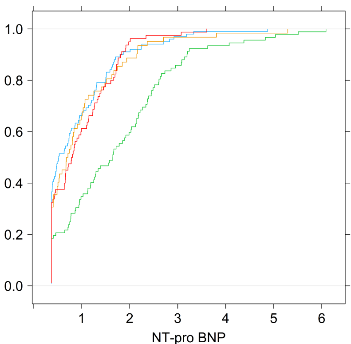

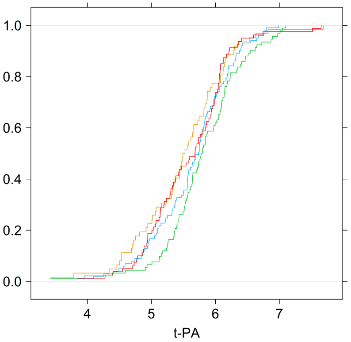

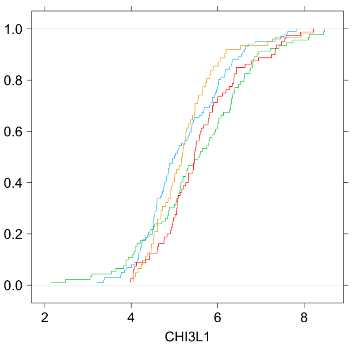
**

**
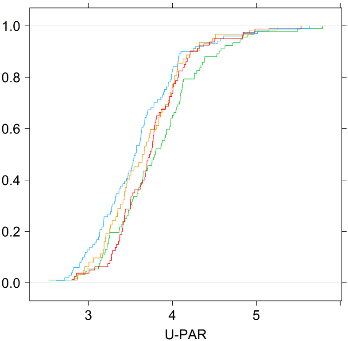

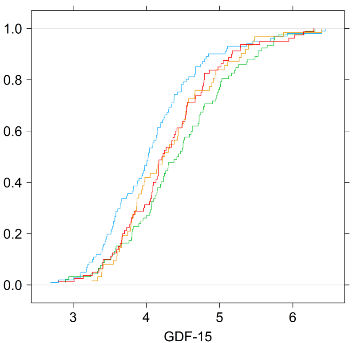

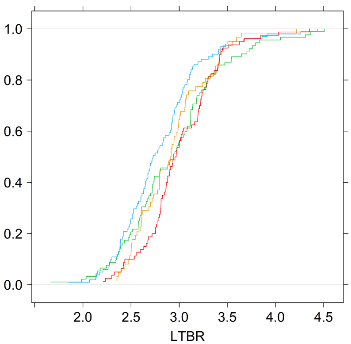
**

**
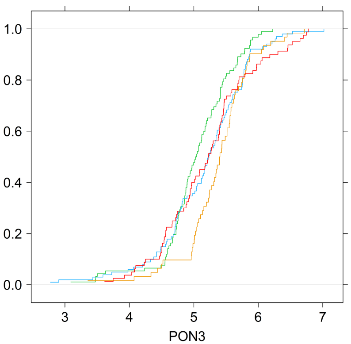

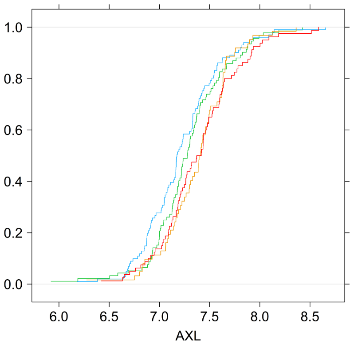

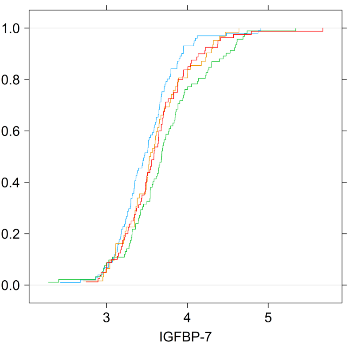
**

**
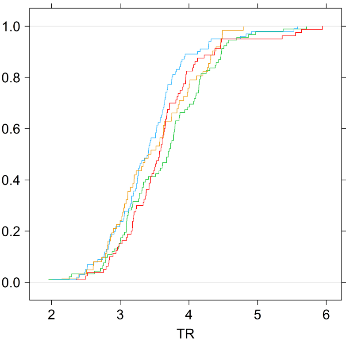
**

t-PA; tissue-type plasminogen activator, CHI3L1; chitinase 3 like protein, U-PAR; Urokinase plasminogen activator surface receptor, GDF 15; growth differentiation factor 15, LTBR; lymphotoxin beta receptor, PON3; paraoxonase 3, AXL; tyrosine kinase receptor UFO, TR; transferrin receptor protein 1

The empirical cumulative distribution function (ECDF) plot describing the distribution of the biomarkers expressed as normalized protein expression (NPX) separated by left ventricular ejection fraction (LVEF) (LVEF) quartiles, ≤53% (green), >53%-≤60% (blue), >60%-≤64% (yellow), and >64% (red), one year after the acute coronary syndrome.

**Figure 6**

**
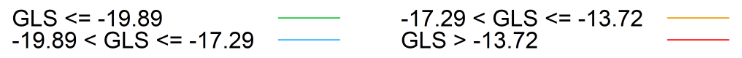
**

**
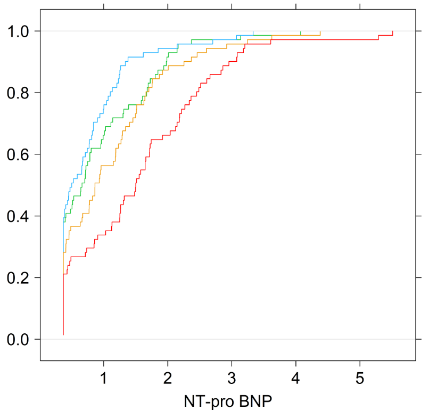

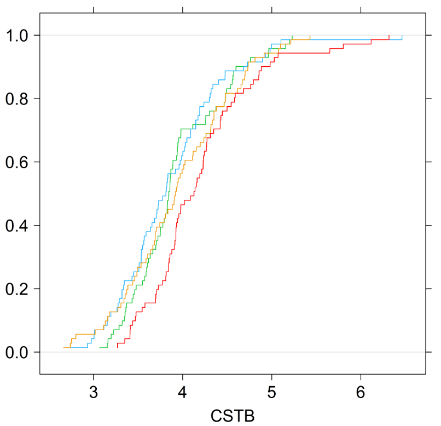

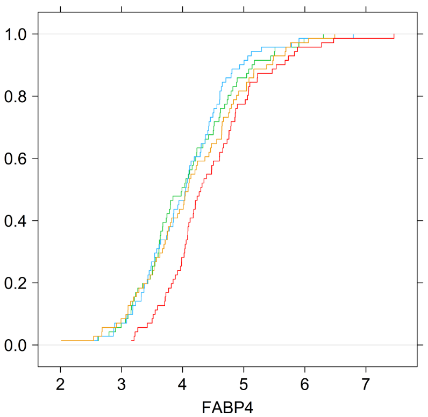
**

**
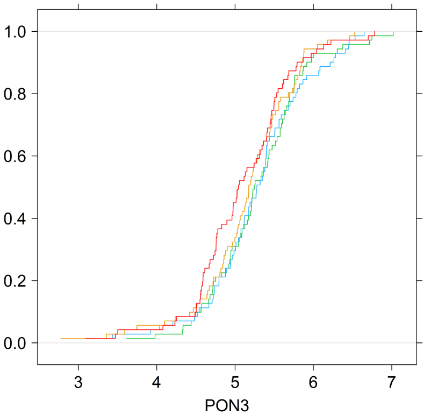

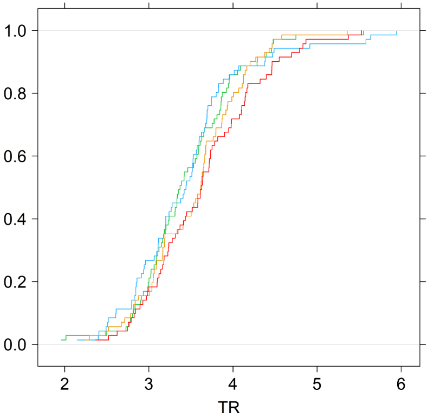

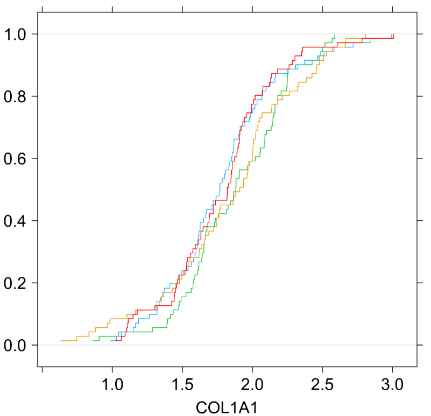
**

**
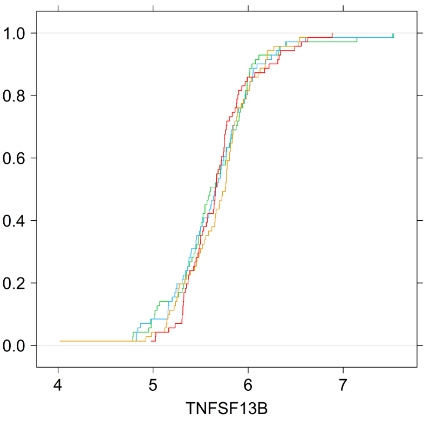

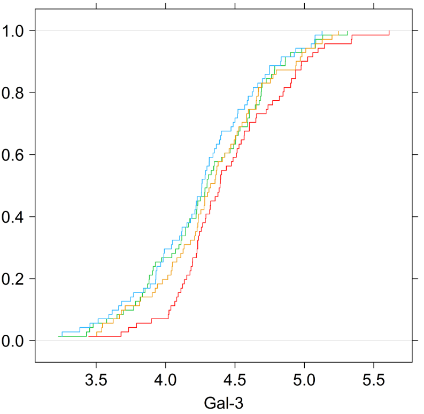

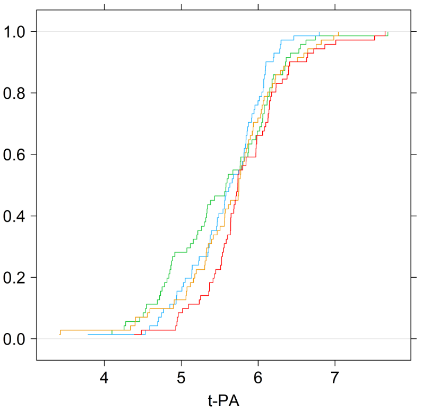
**

**
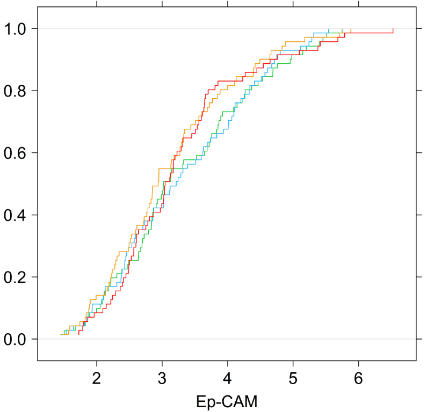
**

CSTB; cystatin-B, FABP; fatty acid-binding protein, adipocyte, PON3; paraoxonase 3, TR; transferrin receptor protein 1¸ COL1A1; collagen alpha 1 chain, TNFSF13B; tumor necrosis factor ligand superfamily member 13B, Gal-3; galectin 3, t-PA; tissue-type plasminogen activator, Ep-CAM; epithelial cell adhesion molecule

The empirical cumulative distribution function (ECDF) plot describing the distribution of the biomarkers expressed as normalized protein expression (NPX) separated by global longitudinal strain (GLS) quartiles, ≤- 19.80 % (green), -19.80- ≤-17.29 % (blue), -17.29-≤-13.72 % (yellow) and > -13.72% (red), one year after the acute coronary syndrome.
